# Bioactive compounds from the parasitic plant *Arceuthobium vaginatum* inhibit *Haemonchus contortus* egg hatching

**DOI:** 10.1590/S1984-29612024004

**Published:** 2023-12-22

**Authors:** María Mitsi Nalleli Becerril-Gil, Julieta Gertrudis Estrada-Flores, Manases González-Cortazar, Alejandro Zamilpa, Ángel Rolando Endara-Agramont, Pedro Mendoza-de Gives, María Eugenia López-Arellano, Agustín Olmedo-Juárez

**Affiliations:** 1 Instituto de Ciencias Agropecuarias y Rurales, Universidad Autónoma del Estado de México, Toluca, Estado de México, México; 2 Centro de Investigación Biomédica del Sur, Instituto Mexicano del Seguro Social, Xochitepec, Morelos, México; 3 Centro Nacional de Investigación Disciplinaria en Salud Animal e Inocuidad – CENID SAI-INIFAP, Jiutepec, Morelos, México

**Keywords:** Arceuthobium, anthelmintic activity, polyphenols, Haemonchus, organic extract, Arceuthobium, atividade anti-helmíntica, polifenóis, Haemonchus, extrato orgânico

## Abstract

The aim of this study was to assess the *in vitro* ovicidal activity of an ethyl acetate extract from *Arceuthobium vaginatum* (EtOAc-E) and their subfractions (AvR5-AvR14) against *Haemonchus contortus* using the egg hatching inhibition (EHI) test. The EtOAc-E and subfractions were tested at 0.12-2.00 and at 0.015-2.0 mg/mL, respectively. Distilled water and methanol (2%) were used as negative controls and Thiabendazole (0.10 mg/mL) as a positive control. Treatments with a dependent effect on concentration were subjected to regression analysis to determine the effective concentrations (EC_50_ and EC_90_). The major secondary compounds present in the extract and subfractions were identified by high performance liquid chromatography (HPLC). The EtOAc-E and AvR9 exhibited the best ovicidal effect recording 97.5 and 100% of EHI at 0.25 mg/mL, respectively. The EtOAc-E and AvR9 displayed an EC_50_= 0.12 and 0.08 mg/mL, respectively. The HPLC analysis in the EtOAc-E and bioactive fractions indicated the presence of a polyphenol, glycosylated flavanones, quercetin glucoside, cinnamates, coumarin, cinnamic acid derivative, ferulic acid, coumarate, naringenin, protocatechuic acid and naringin. Results demonstrated that *A. vaginatum* extract and fraction is able to inhibit the egg hatch process of *H. contortus* and could be a viable option for the control of small ruminant haemonchosis.

## Resumo

• O objetivo deste estudo foi avaliar a atividade ovicida *in vitro* de um extrato de acetato de etila de *Arceuthobium vaginatum* (EtOAc-E) e suas subfrações (AvR5-AvR14) contra *Haemonchus contortus*, utilizando-se o teste de inibição da eclosão de ovos (EHI). O extrato foi testado em 0.12-2.00 mg/mL, e as subfrações foram testadas em 0.15-2.0 mg/mL. Água destilada e metanol (2%) foram utilizados como controles negativos e Tiabendazol (0.10 mg/mL) como controle positivo. Os tratamentos com efeito concentração dependente foram submetidos à análise de regressão para determinação das concentrações efetivas (CE_50_ e CE_90_). Os principais compostos secundários, presentes no extrato e nas subfrações, foram identificados por cromatografia líquida de alta eficiência (HPLC). EtOAc-E e subfração AvR9 exibiram o melhor efeito ovicida, registrando-se 97.5 e 100% do EHI a 0.25 mg/mL, respectivamente. O EtOAc-E e AvR9 apresentaram CE_50_= 0,12 e 0,08 mg/mL, respectivamente. A análise por HPLC nas frações EtOAc-E e bioativas indicou a presença de um polifenol, flavanonas glicosiladas, glicosídeo de quercetina, cinamatos, cumarina, derivado de ácido cinâmico, ácido ferúlico, cumarato, naringenina, ácido protocatecuico e naringina. Os resultados demonstraram que extrato e frações de *A. vaginatum* são capazes de inibir o processo de eclosão dos ovos de *H. contortus*, podendo ser uma opção viável para o controle da hemoncose em pequenos ruminantes.

## Introduction

Small ruminant nematodiasis is one of the major diseases that affecting the productivity of farms under grazing conditions causing economic losses (Zajac & Garza, 2020). *Haemonchus contortus* is a gastrointestinal nematode (GIN) considered one of the most pathogenic parasites due to its hematophagous habit and its high prevalence in sheep and goats (Kuiseu et al*.,* 2021). The main approach for controlling the GIN including *H. contortus* is through chemical drugs and their irrational use has trigged an anthelmintic resistance problem worldwide (Herrera-Manzanilla et al., 2017; Baudinette et al., 2022). In this sense, several research works using the integral management for control of GIN like grazing rotation, selection of animals resistant to GIN (Colvin et al*.,* 2021), use of diets rich in protein and energy (Can-Celis et al., 2022), biological control using nematophagous fungi (Mendoza-de Gives et al., 2022) and plant extracts with high content of secondary metabolites have been proposed (Jayawardene et al., 2021). A number of *in vitro* studies with extracts of several family plants have demonstrated an important anthelmintic effect on GIN including to *H. contortus* (Alowanou et al., 2019; Davuluri et al., 2020; López-Rodríguez et al., 2022). *Arceuthobium vaginatum* (Santalaceae) is a parasitic plant, which is widespread in Mexican and Canadian pine trees (Hawksworth et al., 2002; Queijeiro-Bolaños et al., 2013). There are some practices for controlling this parasitic plant such as chemical control and the pruning (Sotero-García et al., 2018). Some farmers located in the Flora and Fauna Protection Area of “Nevado de Toluca”, Mexico use this plant as an unconventional feed for sheep (Hernández Luna et al., 2017). Likewise, this vegetal species is used in Mexican traditional medicine for throat lung pain and rheumatism (UNAM, 2023). The chemical composition of *A. vaginatum* has not been described; however, some *Arceuthobium* genera like *A. oxycedri* and *A. americanum* contain phenolic compounds, which have shown antibacterial and antifungal effects (Zaidi et al., 2006; 2008; Pernitsky et al., 2011). Thus, the objective of the present study was to assess the ovicidal effect of an acetate ethyl extract and its fractions from *A. vaginatum* against *H. contortus* under *in vitro* conditions.

## Material and Methods

### Vegetal material

*Arceuthobium vaginatum* samples were harvested at the Flora and Fauna Protection Area of Nevado de Toluca, located in the central and volcanic axis of Mexico State (19°07´07´´ N, 099°46´53” W). One hundred twenty-one trees infested with the parasitic plant were considered as representative samples, which 50 samples were collected in June-August 2021. The mistletoe sample was identified using taxonomic keys available in specialized literature (Hawksworth & Wiens, 1972; Rzedowski et al., 2005) at the facilities of the Instituto de Ciencias Agropecuarias y Rurales of the Universidad Autónoma del Estado de México. It has also been reported that in the Flora and Fauna Protection Area of Nevado de Toluca only the specie *A. vaginatum* subsp. *vaginatum* is distributed (Endara-Agramont et al., 2022). The vegetal material was dehydrated at 35 °C in a botanical dryer for 72 h. Then, the dried plant was ground in a Willey mill to reduce the size particle to 1 mm.

### Obtaining the ethyl acetate extract and subfractions

A representative sample of *A. vaginatum* (2.5 kg) was macerated using 5,000 mL of ethyl acetate at room temperature (25-30 °C) for 24 h. The liquid extract was filtered using different sieves (gauze, cotton and Whatman® 4 filter paper). The liquid ethyl acetate extract (EtOAc-E) was concentrated by distillation under reduced pressure using a rotary evaporator (Buchi R-210, Flawil, Switzerland). Solvent traces were eliminated through a Freeze dryer (Labconco 4.5), resulting in a dark violet solid extract. A part of EtOAc-E (25.3 g) was adsorbed in silica gel (41.8 g normal phase, Merck, Darmstadt, Germany), applied to a glass column with silica gel (4.5 x 65 cm) and eluted with hexane/ethyl acetate with 10% ascendant polarity, collecting 36 fractions of 200 mL each. These fractions were concentrated in a rotary evaporator under the same conditions previously described and grouped according to their similarity by thin layer chromatography (TLC) into 14 subfractions (AvR1-AvR14). The high yielding subfractions (AvR5, AvR7, AvR9, AvR11, AvR13 and AvR14) were considered for their anthelmintic evaluation.

### Identification of major compounds

The EtOAc-E and subfractions were subjected to chemical analysis by high performance liquid chromatography (HPLC) using a Waters 2695 separation module system equipped with a Waters 996 photodiode array detector and Empower Pro software (Water Corporation, USA). Chemical separation was achieved using a Supelcosil LC-F reverse phase column (250 mm x 4.6 mm i.d., 5 µm particle size, Sigma-Aldrich, Bellefonte, USA). The mobile phase consisted of 0.5% trifluoroacetic acid aqueous solution (Solvent A) and acetonitrile (Solvent B). The gradient system was as follows: 0-1 min 0% B; 2-3 min, 5% B; 4-20 min, 30% B; 21-23 min 50% B; 24-25 min, 80% B; 26-27 min, 100% B; 28-30 min, 0% B. The flow rate was maintained at 0.9 mL/min, and the sample injection volume was 10 µL. Absorbance was measured at 330 nm. The identification of compounds was performed based on a direct comparison of the retention times and UV spectra with the reference standards (Zarza-Albarrán et al., 2020).

### Collection of *Haemonchus contortus* eggs

The *H. contortus* eggs were obtained from an egg-donor lamb (20 kg of bodyweight) previously infected with 7,000 *H. contortus* L_3_ (INIFAP-HcIVMr-SAI strain, Mexico). This isolate was obtained from a naturally infected grazing sheep from a tropical region in the Salto de Agua municipality, Chiapas, Mexico (González-Garduño et al., 2012; Reyes-Guerrero et al., 2023). The lamb was housed indoors on a metabolic floor, feed hay and commercial concentrate and had free access to water. The animal was housed following the care/welfare guidelines of the Mexican Official Rule NOM-051-ZOO-1995 (México, 1999). Egg recovery was performed according to the technique described by Coles et al. (1992) with minor modifications. Briefly, 50-60 g of faeces were collected directly from the rectum of the animal. The faecal material was macerated with clean water in a mortar and was deposited in Falcon tubes (30 mL faecal solution) to which saline solution (25 mL) was added. The tubes were centrifuged at 3,500 rpm for 5 min and the supernatants were recovered and rinsed with clean water on two sieves of 75 and 32 µm. The *H. contortus* eggs retained in the 32 µm sieve were recovered in a Falcon tube (12 mL) and rinsed three times by centrifugation (3,500 rpm for 3 min) using distilled water. Finally, the *H. contortus* eggs concentration was estimated by counting the number of eggs in ten 5-µL aliquots using an optical microscope until reach a quantity of 100 ± 10 *H. contortus* eggs in 50 µL.

### Egg hatching inhibition (EHI) test

The bioassays were performed in 96-well microtitration plates, and for each treatment four repetitions were performed (n=12). The treatments were assigned as follows: ethyl acetate extract (EtOAc-E at 0.12, 0.25, 0.50, 1.00 and 2.00 mg/mL final concentration), subfractions (AvR5, AvR7, AvR9, AvR11, AvR13 and AvR14) at 0.25, 0.50, 1.00 and 2.00 mg/mL final concentration. The subfraction with the best ovicidal activity (AvR9) was tested at 0.015-2.00 mg/mL. Additionally, distilled water and 2% methanol (to solubilized the extract and subfractions) were used as negative controls and Thiabendazole (0.1 mg/mL) as a positive control. An aqueous suspension of 50 µL containing 100 ± 10 *H. contortus* eggs was deposited in each well. Then, 50 µL of treatments were deposited in each well. The plates were incubated at room temperature (25-30 °C) for 48 h. Following incubation, the total eggs or larvae (L1 and L2) of each well were counted under an optical microscope at 10^x^. The EHI percentage for each treatment was determined according to the following Formula 1:


$$\%\;EHI=\left[\left(number\;of\;eggs\right)/\left(number\;of\;larvae+number\;eggs\right)\right]\times 100$$ (1)

### Statistical analysis

The EHI percentages were analysed based on a completely randomised design using ANOVA through a general linear model with the following statistical model: Y*_ij_* = μ + T*_i_* + ξ*_ij_*; where Y*_ij_*= response variable (EHI%), μ= general mean and ξ*_ij_*=error. The differences among treatments were compared with a Tukey’s test (*P* < 0.05). The concentration-effect dependent treatments were subjected to regression analysis to determine the effective concentrations (EC_50_ and EC_90_), using the Proc Probit analysis in SAS 9.0 (SAS Institute, 2006).

## Results

### HPLC analysis of the chemical constituents of the ethyl acetate extract and fractions

**Figure 1:**
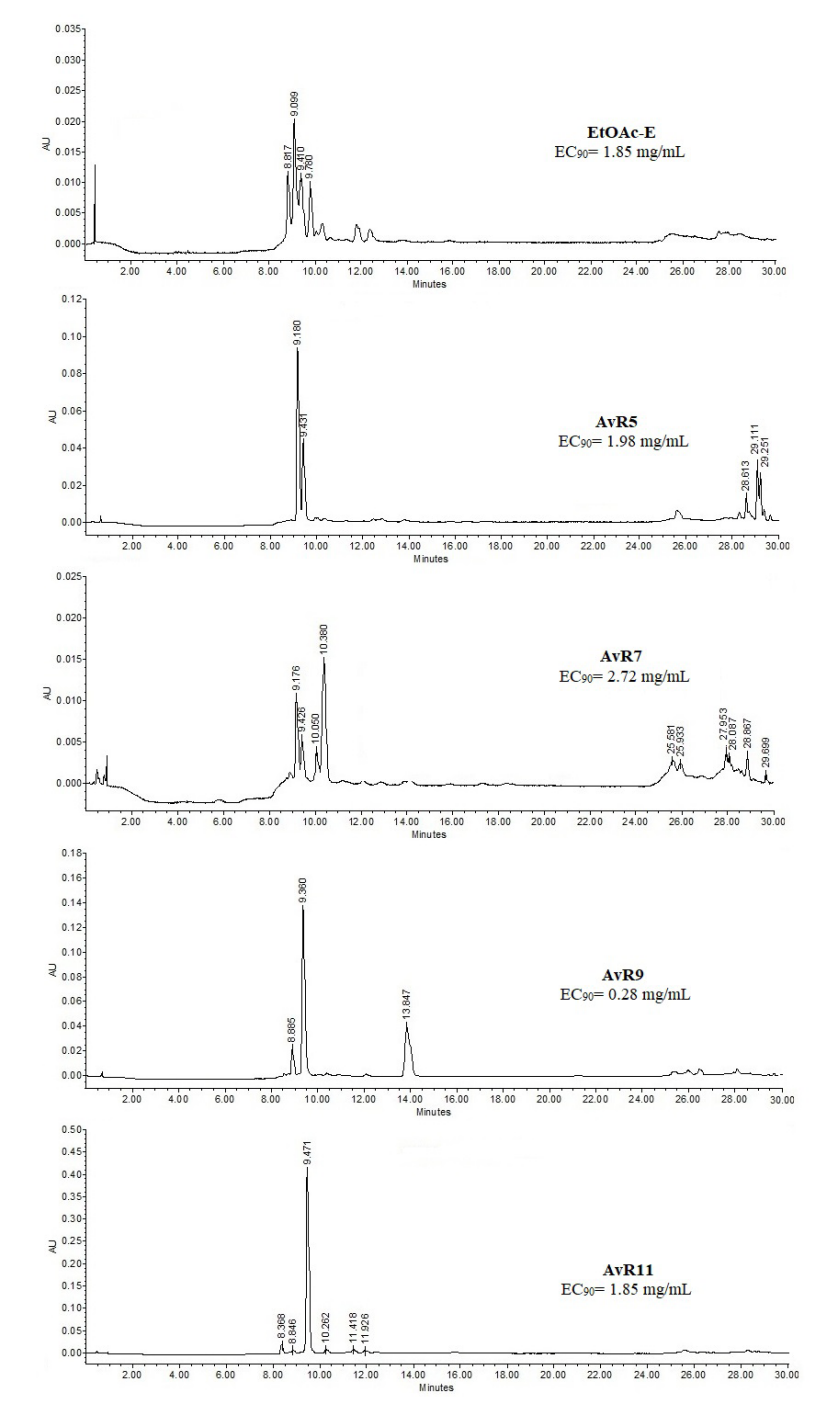
HPLC analysis of *Arceuthobium vaginatum* showing egg-hatching inhibition of *Haemonchus contortus* eggs. EC = effective concentration.

The HPLC chromatograms of the EtOAc-E and its subfractions are shown in Figure 1. According to retention times (Rt) and the UV absorption spectra (λ_max_), the identified compounds in the EtOAc-E were a polyphenol (Rt= 8.81 min, λ_max_ = 191.1, 229.8 and 360.6 nm), two glycosylated flavanones (Rt= 9.09, λ_max_ = 206.3, 287.6 and 343.4 nm; Rt= 9.41 min, λ_max_ = 208.7, 288.8 and 427.1 nm) and quercetin glucoside (Rt= 9.78 min, λ_max_ = 194.6, 256.9 and 355.3). The AvR5 showed the presence of cinnamates (Rt= 9.18, λ_max_= 221.6 and 285.2 nm; Rt= 29.1 min, λ_max_= 192.3 and 280.5 nm; Rt= 29.25 min; λ_max_= 198.2 and 280.5 nm), a coumarin (Rt= 9.43 min, λ_max_= 231.0, 279.3 and 310.1 nm) and a flavone (Rt= 28.61 min, λ_max_= 211.0, 254.5 and 304.2 nm). The major compounds identified in the AvR7 subfraction were cinnamic acid derivative (Rt= 9.17 min, λ_max_=223 and 284 nm) and ferulic acid (Rt= 10.38 min, λ_max_ =233.3 and 325.5 nm). The best bioactive fraction (AvR9) showed the presence of a coumarate (Rt= 9.36 min, λ_max_= 192.3, 228.6 and 310.1 nm) and naringenin (Rt= 13.85 min, λ_max_= 213.4 and 289.9 nm). Regarding AvR11, protocatechuic acid (Rt= 8.36 min, λ_max_=214.5, 260.4 and 294.7 nm) and naringin (Rt= 9.47 min, λ_max_=213.4 and 288.8 nm) were observed.

### Egg hatching inhibition (EHI) test

The *H. contortus* EHI percentages caused by EtOAc-E and subfractions, as well as their proper controls, are shown in Table 1. The EtOAc-E displayed an ovicidal effect greater than 90% from at 0.25 mg/mL concentration. The AvR9 subfraction displayed the best ovicidal activity (P<0.05), reaching a 96.10% EHI from 0.25 mg/mL. Subfractions AvR5, AvR7 and AvR11 showed an EHI greater than 80% at 2.00 mg/mL. Meanwhile, AvR13 and AVR14 recorded the lowest ovicidal effect in the range of 17.28-36.29% EHI.

**Table 1:** *Haemonchus contortus* egg hatching inhibition percentages (EHI%) of an ethyl acetate extract (EtOAc-E) and subfractions from *Arceuthobium vaginatum*.

**Treatments**	**Means of eggs and larvae (L1 or L2) recovered**		**EHI% ± s.d.**
	**Eggs**	**Larvae**	
**Thiabendazole (0.1 mg/mL)**	93.2	0.2	99.6 ± 0.8^ab^
**Distilled water**	11.8	77.0	13.8 ± 8.6^g^
**Methanol 2%**	13.0	81.2	14.8 ± 9.2 ^g^
**EtOAc-E, mg/mL**			
**0.12**	23.1	58.3	29.2 ± 5.7^efg^
**0.25**	111.1	5.8	94.5 ± 6.3^abc^
**0.50**	117.8	4.2	96.5 ± 4.2^abc^
**1.00**	117.2	2.8	97.6 ± 1.5^ab^
**2.00**	107.5	1.9	97.4 ± 3.9^abc^
**AvR5, mg/mL**			
**0.25**	30.0	75.0	26.8 ± 8.3^efg^
**0.50**	23.0	74.3	22.4 ± 5.5^efg^
**1.00**	76.1	32.7	69.3 ± 21.0^d^
**2.00**	102.2	10.3	89.4 ± 9.0^abc^
**AvR7, mg/mL**			
**0.25**	23.1	76.1	23.0 ± 4.4 ^efg^
**0.50**	28.5	71.2	28.3 ± 4.4^efg^
**1.00**	42.4	57.5	37.6 ± 13.3^e^
**2.00**	85.3	15.1	83.0 ± 15.2^abcd^
**AvR9, mg/mL**			
**0.015**	13.5	67.0	17.7 ± 6.3^g^
**0.03**	16.2	60.0	18.5 ± 9.8^g^
**0.06**	20.5	75.1	22.4 ± 5.6^efg^
**0.12**	89.8	19.6	80.4 ± 11.9^cd^
**0.25**	131.0	5.0	96.1 ± 2.5^abc^
**0.50**	95.9	5.4	91.0 ± 9.8^abc^
**1.00**	104.2	1.8	96.2 ± 5.6^abc^
**2.00**	126.5	0.0	100.0^a^
**AvR11, mg/mL**			
**0.25**	18.2	80.2	17.7 ± 9.3^g^
**0.50**	20.6	64.6	22.6 ± 15.4^efg^
**1.00**	87.1	15.0	82.5 ± 21.4^bcd^
**2.00**	95.6	12.1	88.0 ± 11.2^abc^
**AvR13, mg/mL**			
**0.25**	20.0	96.0	17.2 ± 3.6^g^
**0.50**	22.5	93.0	19.4 ± 3.4^fg^
**1.00**	17.5	84.5	18.5 ± 5.8^g^
**2.00**	28.5	74.5	28.5 ± 8.8^efg^
**AvR14, mg/mL**			
**0.25**	18.1	75.4	20.5 ± 7.2^efg^
**0.50**	21.6	76.8	23.2 ± 7.5^efg^
**1.00**	27.6	66.5	30.2 ± 7.1^efg^
**2.00**	32.5	58.1	36.2 ± 6.2^ef^
**Variation coefficient**			17.29
**R^2^**			0.94

**abcdefg:** Means with different literal in the same column indicate statistical differences (P<0.05). s.d. = standard deviation; AvR5-AvR14 = subfractions obtained from EtOAc-E.

### Effective concentrations 50 and 90

The effective concentrations (EC) required to cause 50 and 90% egg-hatching inhibition of EtOAc-E and its subfractions are shown in Table 2. The EtOAc-E and AvR9 induced 50% inhibition at minimum concentration (EC_50_=0.12 and 0.088 mg/mL) compared with to other subfractions.

**Table 2:** Effective concentrations required to cause 50% and 90% of *Haemonchus contortus* egg hatching inhibition after 48 h exposure to an ethyl acetate extract and its bioactive fractions from *Arceuthobium vaginatum*.

Treatments	EC_50_ mg/mL	Confidential interval (95%)		EC_90_ mg/mL	Confidential interval (95%)	
		Lower	Upper		Lower	Upper
EtOAc-E	0.12	0.11	0.13	0.42	0.39	0.45
AvR5	0.91	0.86	0.96	1.98	1.85	2.15
AvR7	1.17	1.09	1.24	2.72	2.49	3.05
AvR9	0.088	0.083	0.094	0.28	0.26	0.29
AvR11	0.79	0.75	0.83	1.85	1.73	1.99

EC = effective concentration; EtOAc-E = ethyl acetate extract; AvR5-AvR7 = subfractions obtained from EtOAc-E.

## Discussion

### Egg hatching inhibition (EHI) test

The results obtained in the present study show that *A. vaginatum* EtOAc-E is able to inhibit the egg-hatching process of *H. contortus*. The chemical fractionation of the integrate extract allowed obtain some subfractions with important inhibition effects against this parasite. A number of studies, under *in vitro* and *in vivo* conditions using organic plant extracts with different polarity on nematodes have evidenced nematocidal effects (Morais-Costa et al., 2016; Mengistu et al., 2017; Badar et al., 2021; Hassan et al., 2021). The ability of solvents to extract secondary compound groups in several plant families (Fabaceae, Asteraceae, Bixaceae, Santalaceae, etc.) has been documented worldwide (Akkol et al., 2010; Zeb et al., 2017; Ondua et al., 2021). Studies have been conducted with extracts of intermediate polarity like acetonic or with ethyl acetate, which have demonstrated a potent ovicidal and larvicidal effect against gastrointestinal nematodes including *H. contortus*. For instance, Mhomga et al. (2022) tested an acetonic extract from *Cochlospermum planchonii* (Bixaceae) on *H. contortus eggs* and reported an ovicidal effect of 100% at 0.31 mg/mL. These results were similar to the findings of our study with the *A. vaginatum* EtOAc-E. In another study with an EtOAc-E and two fractions from *Ananas comosus* (Bromeliaceae), an ovicidal effect close to 100% at 5 mg/mL on *H. contortus* eggs was recorded (Rodrigues et al., 2020). In the present study, when the extract was fractionated, the subfraction AvR9 exhibited the same ovicidal effect at 0.25 mg/mL (Table 1).

*Arceuthobium vaginatum* is used in traditional medicine in Mexico to treat some respiratory and gastrointestinal diseases. According to the available literature and to our knowledge, there are no reports about this plant concerning anthelmintic properties. Thus, this will be the first report of the nematicidal activity of *A. vaginatum* against *H. contortus*. There are reports about some plants belonging to the Santalaceae family with anthelmintic effect. For instance, Payne et al. (2013) found an important nematocidal effect of an aqueous extract from *Santalum spicatum* against cyathostomins, nematodes with a high prevalence in horses. In another study by Tibe et al. (2013) it was demonstrated that the ethyl acetate extract from *Viscum rotundifolium* inhibited the larval development of *H. contortus* at 100 µg/mL. In this same study, the authors did not found egg-hatching inhibition in this extract.

Analyzing the EC_50_ results of the present study, the AvR9 subfraction was 13.29, 10.34 and 1.36 more effective than the AvR7, AvR5 and AvR11 subfractions and the EtOAc-E. The *A. vaginatum* EtOAc-E was more active than those reported with an extract with the same polarity from *Typha capensis* (EC_50_= 0.12 *vs* 0.43 mg/mL) on *H. contortus* eggs (Ondua et al., 2021) and *Ananas comosus* (EC_50_= 0.45 mg/mL) (Rodrigues et al., 2020). In our study, the concentration required to cause a 50% of ovicidal effect from *A. vaginatum* EtOAc extract was only 0.12 mg/mL (Table 2). These differences could be related to the family/species used, since the amount and type of bioactive compounds contained in *A. comosus* are different in comparison to *A. vaginatum*.

### Major compounds identified by HPLC analysis

The chemical fractionation of *A. vaginatum* EtOAc extract allowed us to identify the hydroxycinnamic acid derivatives, coumarates and the flavonoid naringenin. According to the literature, this plant contains flavonoids such as quercetin-3-O-galactoside, myricetin-3-O-galactoside and quercetin-3-O-glycoside (Crawford & Hawksworth, 1979). In other mistletoe genus (*Viscum* spp.), flavonoids, alkaloids and saponins were identified (Hawu et al., 2022). Compounds belonging to hydroxycinnamic acid derivatives like coumaric acid, ferulic acid, caffeic acid and chlorogenic acid are phytochemicals present in the fruits and aerial pars of several plants, which are known to exert beneficial effects linked to their antioxidant and anthelmintic activity (Mancilla-Montelongo et al., 2019; Ondua et al., 2021; Orzuna-Orzuna et al., 2023). Some bioguided studies with plants rich in secondary metabolites have identified molecules like hydroxycinnamic acid and naringenin. For example, the coumaric acid has been isolated from *A. comosus* and was evaluated against *H. contortus*, demonstrating an important nematicidal effect (Rodrigues et al., 2020). Thus, the ovicidal activity of AvR5 and AvR7 could be attributed to the hydroxycinnamic acid present in these fractions. Several reports assessing phenolic compounds in combined form have evidenced that they can enhance the anthelmintic effect (Klongsiriwet et al., 2015; von Son-de Fernex et al., 2015; Mendonça Soares et al., 2019). Chlorogenic acid and chatequin have been reported in *A. oxycedri*, another mistletoe specie (Orhan et al*.,* 2019). Chlorogenic acid was isolated from *Tagetes filiflora* (Asteraceae) and showed 100% of EHI at 0.5 mg/mL against *H. contortus* (Jasso Díaz et al., 2017). Another phenolic acid like coumaric acid also showed ovicidal and larvicidal effects on this parasite (Castañeda-Ramírez et al., 2019; Mancilla-Montelongo et al., 2019; Rodrigues et al., 2020). In this regard, these chemical constituents present in *A. vaginatum* could be responsible for ovicidal activity. Another compound identified in the AvR9 subfraction was naringenin, which has been associated with an anthelmintic effect on *H. contortus* (Zarza-Albarrán et al., 2020). The naringenin together with the coumarate present in the AvR9 subfraction might act in a synergic form on the *H. contortus* eggs.

On the other hand, the anthelmintic effect of some coumarins has been documented, i.e., von Son-de Fernex et al. (2017) evaluated the coumarin 2H-chromen-2-one from *Gliricidia sepium* leaves (Fabaceae) on *Cooperia punctata*, a nematode of cattle and observed that this compound inhibited the egg hatching process. In our study, the presence of coumarins, cinnamates and flavones was observed in the AvR5 subfraction. These compounds present in this fraction could act in a synergic form on the eggshell of the parasite, which interrupts the development of the next stage. There is information about the possible mechanism of action of some phenolic compounds on the eggs or larvae of gastrointestinal nematodes. The anthelmintic activity of phenolic compounds could be associated with the enzymatic process of parasites; the exchange of these compounds with the *H. contortus* eggshell structures could provoke inhibition of the development of the eggs to the following stage (Rabbi et al., 2020; Ondua et al., 2021). The interaction of some phenolic compounds with the egg membrane causes structural changes affecting its permeability, oxygen exchange, and release of substances and enzymes involved in eggshell degradation and this allows no release of the larvae (Rogers & Brooks, 1977; Vargas-Magaña et al., 2014; Engström et al., 2016). Recent studies with confocal laser microscopy scanning analysis have demonstrated important scientific evidence concerning the possible mechanism of action of the flavonol isokaempferide and coumaric acid on the *H. contortus* egg hatching process, where there is colocalization of the compounds with the egg membrane (Cortes-Morales et al., 2019, 2022). These findings indicate that these compounds pass through the external cuticle of the eggs and there is a chemical interaction between the compounds with the *H. contortus* morula or embryo (Cortes-Morales et al., 2022). This evidence could help to knowledge of the interaction of the secondary compounds with the parasites. Unfortunately, in the present study microscopic analysis of the bioactive subfractions on *H. contortus* eggs was not performed and further studies will be considered to corroborate such interaction.

In the present study, it was demonstrated that the EtOAc extract from *A. vaginatum* contains bioactive compounds that interrupt the life cycle of *H. contortus*. The coumarate and naringenin present in the AvR9 subfraction revealed the best ovicidal activity against this parasite and could represent a candidate for use in further *in vivo* studies. Additionally, chemical characterization of the extract is necessary to determine the identity and amount of the compounds responsible for the anthelmintic activity.
